# Leptin Promotes Glioblastoma

**DOI:** 10.1155/2012/870807

**Published:** 2012-01-04

**Authors:** Johnathan E. Lawrence, Nicholas J. Cook, Richard A. Rovin, Robert J. Winn

**Affiliations:** ^1^Biology Department, Northern Michigan University (NMU), Marquette, MI 49855, USA; ^2^Upper Michigan Brain Tumor Center, NMU and Marquette General Hospital, Marquette, MI 49855, USA; ^3^Neurosurgery, Marquette General Hospital, Marquette, MI 49855, USA

## Abstract

The hormone leptin has a variety of functions. Originally known for its role in satiety and weight loss, leptin more recently has been shown to augment tumor growth in a variety of cancers. Within gliomas, there is a correlation between tumor grade and tumor expression of leptin and its receptor. This suggests that autocrine signaling within the tumor microenvironment may promote the growth of high-grade gliomas. Leptin does this through stimulation of cellular pathways that are also advantageous for tumor growth and recurrence: antiapoptosis, proliferation, angiogenesis, and migration. Conversely, a loss of leptin expression attenuates tumor growth. In animal models of colon cancer and melanoma, a decline in the expression and secretion of leptin resulted in a reduction of tumor growth. In these models, positive mental stimulation through environmental enrichment decreased leptin secretion and improved tumor outcome. This review explores the link between leptin and glioblastoma.

## 1. Introduction

Leptin is the product of the *obese* gene, located on chromosome 7 in humans. Mice with mutation in the *obese* gene are obese and insatiable [1]. When exogenous leptin is injected into leptin-deficient obese mice (ob/ob mice), the protein promotes satiety and weight loss [2–5]. The effects of leptin on these obese mice sparked a leptin intense focus in obesity research over the past 15 years. Unlike the ob/ob mice, obese humans are not leptin deficient. Obese humans have high circulating leptin levels which are directly correlated to the total amount of adipose tissue [6]. Leptin helps regulate bodyweight in humans by negative feedback promoting satiety when energy stores are elevated [7]. The current model suggests that obesity in humans is due to a desensitization to leptin. Obese subjects have a diminished response to leptin, and in some subjects the diminished response is due to a mutation in the leptin receptor gene [8]. The high prevalence of obesity in the USA is strongly correlated with the risk of multiple diseases, including cancer [9]. The association between cancer and obesity may, in part, be explained by elevated circulating leptin.

## 2. Leptin in Cancer

**Figure 1:**
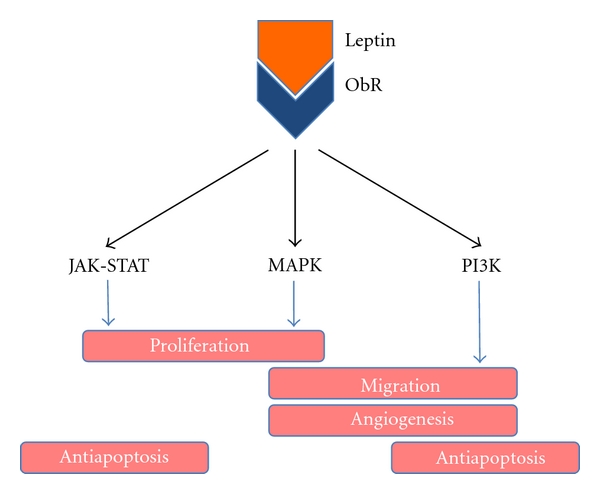
Cellular pathways activated through leptin receptor (ObR) stimulation.

Leptin has been classified as a growth factor because it stimulates three key pathways well known for their roles in cell growth: proliferation, survival, and motility and migration (Figure 1). It is well documented that the binding of leptin to the leptin receptor (ObR) activates the Janus kinase-signal transducer and activator of transcription (JAK-STAT), the mitogen-activated protein kinase (MAPK), and the phosphatidylinositol 3-kinase (PI3K) pathways in both normal [10–22] and malignant cells [20, 23–41]. Supporting a role of leptin in cancer pathogenesis are reports that DNA polymorphisms in the leptin and ObR genes are associated with increased risk and progression of breast [42], prostate [43], and oral cancer [44].

Evidence generally supports leptin as a growth factor, promoting cell division and evasion of cell death [45]. Numerous reports indicate that leptin has both antiapoptotic [28, 33, 34, 36, 46–54] and proliferative effects [24, 25, 27, 29–31, 33, 34, 36, 41, 47, 49, 50, 52, 53, 55–59] (Table 1). It appears that leptin-mediated proliferation of these cancers occurs through the activation of the JAK-STAT [25, 27, 29–31, 34, 41], PI3K [24, 31, 33, 36], and MAPK [24, 31] pathways, whereas apoptosis avoidance is promoted by leptin via the JAK-STAT [28, 34] and PI3K [33, 36] pathways (Figure 1).

Migration is enhanced by leptin in several normal [10, 20–22, 60, 61] and cancerous tissues [20, 23, 26, 32, 35, 37, 39, 40, 62–64] (Table 1). Leptin treatment increases the growth and migration of cholangiocarcinoma cells *in vitro* and cholangiocarcinoma is inducible in obese fa/fa Zucker (faulty ObR) rats [53]. In metastatic colon cancer cells, leptin provokes the formation of lamellipodia and augments invasion through the MAPK and PI3K pathways [62]. It has since been confirmed that leptin increases migration through the MAPK and PI3K pathways in prostate [37, 39, 40], liver [26], cartilage [32], and breast [23, 40, 64] cancers, as well as the JAK-STAT pathway in colon [35], prostate [39], liver [26], and breast [23] cancers. Compounding the complexity of leptin's role in carcinogenesis is that leptin may have differential responses in closely related cells; leptin induces migration in papillary thyroid cancer cells but not in anaplastic and follicular thyroid cancer cells [63].

In addition to its role in cellular proliferation, apoptosis avoidance, and migration, leptin is a potent angiogenic factor. Using an *in vitro* angiogenesis assay, leptin enhances the formation of capillary-like tubes by human umbilical venous endothelial cells [65]. In 5- to 6-week-old C57BL/6J mice, leptin induces fenestrated blood vessel growth [66]. This response is synergistic with vascular endothelial growth factor (VEGF) and fibroblast growth factor-2 [66]. Myometrial cells and the blood-vessel walls of uterine myomas contain leptin, though the surrounding normal tissue does not. This suggests that leptin may be involved in angiogenesis and the development of uterine cancer [67]. VEGF levels are augmented by leptin in various cancers [37, 38, 58, 68]. It has been reported that the leptin-induced upregulation of VEGF may be due to activation of the IL-1 system [38]. This leptin-mediated IL-1 up-regulation appears to be accomplished by activation of the MAPK and PI3K pathways, among others [37, 38]. Leptin and ObR expression are correlated with the grade of the tumor, differentiation, and microvessel density [58, 69]. VEGF expression is also correlated to these variables [58]. It is noteworthy to mention that Per Ole Iverson and coworkers blocked the ObR which suppressed rat leukemia cell growth by inhibiting angiogenesis [70]. Interestingly, hypoxia can induce VEGF production in cells, and it has been demonstrated that leptin expression is also augmented under similar conditions [71].

## 3. The Leptin GBM Connection

It was once thought that adipocytes were the sole producers of leptin. However, leptin expression and secretion has since been demonstrated in several tissues of the body (cancerous and noncancerous) including the pituitary gland and hypothalamus [72]. Barbara Morash and colleagues provided the first report of leptin expression in glioma following detection of leptin expression in the rat C6 glioma cell line [72]. It was later shown that C6 cells express more leptin and ObR than normal glial tissue [73]. Leptin and ObR expression subsequently has been confirmed in human primary GBM tissue as well as established human GBM cells lines [74]. Leptin and ObR are overexpressed in human primary brain tumors when compared to normal glial tissue [74]. Furthermore, the expression of the leptin-ObR system correlates with histological grade: GBM has the greatest levels of leptin and ObR while low-grade gliomas have the least [74]. This suggests that leptin/ObR autocrine/paracrine signaling increases the malignant characteristics of gliomas.

Leptin/ObR overexpression in glioma [74], coupled with recent evidence that the release of leptin from adipose tissue promotes melanoma and colon cancer [75], provides strong evidence that leptin plays a role in cancer pathogenesis. In the rat C6 cell line, leptin knockdown using RNA interference produced a reduction of both leptin mRNA and leptin protein. This knockdown caused a twofold increase in cell death suggesting that endogenous leptin promotes cell survival [76]. Furthermore, exogenous leptin enhances migration and invasion of the rat C6 cells through increased levels of matrix metalloproteinase-13 (MMP-13) [73]. The leptin-mediated up-regulation of MMP-13 occurs through the MAPK pathway [73].

While there is increasing evidence of leptin's role in angiogenesis [37, 38, 58, 68], no studies (to our knowledge) have indicated how leptin might affect angiogenesis in GBM. However, hypoxia, which is a characteristic of solid tumors, is more pronounced with higher grades of glioma [77] and may explain the increased expression of leptin and ObR in GBM compared to lower-grade glioma [74].

## 4. Environmental Enrichment Modulates Leptin Levels

It is increasingly evident that the enhanced mental stimulation from environmental enrichment (EE) delays the advancement of neurodegenerative disorders such as Huntington's, Parkinson's, and Alzheimer's [78], slows the progression of cancer [75, 79–81], and increases the activity of natural killer cells [82]. Environmental enrichment refers to the living conditions of the subject. In the context of the rodent, EE is achieved through conditions that allow the rodent to roam more freely, engage with the surroundings, be housed with other rodents, and have better access to exercise equipment. For humans, increased social and physical activity leads to EE. Interestingly, EE can reduce peripheral leptin expression and release [75].

The response to EE is related to the type of stress the subject experiences: EE increases eustress and decreases distress. Eustress is the result of positive stressors like exercise and social interaction whereas distress is the result of negative stressors like mental stress and social isolation. The augmentation of eustress and the reduction of distress are associated with longer survival and slower tumor growth [75, 79, 81]. Probably the most significant human data to date are those reported by Barbara Andersen and her colleagues who showed that distress reduction through psychological intervention resulted in a 45% decrease in the risk of breast cancer recurrence [79] and a 59% reduction in the risk of dying following breast cancer recurrence [81]. The physiological basis for this finding is an active area of investigation. Using mouse models for melanoma and colon cancer, Cao et al. demonstrated that EE enhances brain-derived neurotrophic factor (BDNF) expression [75]. BDNF in turn activates sympathetic nerve fibers innervating white adipose tissue. This beta-adrenergic stimulation suppresses leptin secretion resulting in cancer inhibition and remission [75].

## 5. Environmental Enrichment and GBM

A study has yet to be designed that blocks ObR or alters leptin levels in GBM subjects or animal models. One viable option for GBM treatment may be through EE. Recall that EE-induced activation of the brain-adipocyte BDNF/leptin axis causes cancer remission and inhibition in mice [75], and distress reduction lowers the rate of recurrence in breast cancer patients [79]. Environmental enrichment and psychological treatment increase BDNF and thereby reduce systemic leptin via sympathetic activation of beta-adrenergic receptors in adipose tissue. This hypothalamic-sympathoneuronal-adipocyte axis does not address the potential leptin-ObR autocrine signaling loop of GBM. Factors that influence the transcriptional regulation of the leptin gene in the rat C6 cells are different than those in adipose tissue [83, 84], and therefore successful treatments may need to be more specific to GBM. Therapies that are successful at crossing the blood-brain barrier and reducing the leptin-ObR signaling loop in GBM are needed and should be a focus of future research.

## 6. Summary

Leptin, which may be controlled by specific stimulation of the brain via EE or psychological intervention, has significant influence on tumor growth. In GBM and other cancer cells, leptin promotes cancer by stimulating cellular pathways that are advantageous for proliferation, angiogenesis, and evasion of death. Unfortunately, most of what is known about leptin and glioma stems from the rat C6 cell line. Future studies should focus on established human GBM cell lines and primary GBM neurosphere cultures both *in vitro* and *in vivo*.

## Figures and Tables

**Table 1:** Summary of the Literature: leptin's role in cancer promotion*.

Cancer type	Antiapoptosis	Proliferation	Migration	Angiogenesis
Bone		24		
Breast	28, 46	27, 56	23, 65	38, 69
Cartilage			32	
Colon	48	57, 58	20, 62, 64	58
Endometrial	34	30, 31, 34		
Esophageal	51			
Gallbladder		53	53	
Gastric		25, 59		
Glioma	77		74	
Kidney		29		
Large B-cell lymphoma	33			
Leukemia	47	47		71
Liver	52	41, 52	26	70
Lung	53			
Neuroblastoma	49	49		
Ovarian		55		
Prostate	50	50	37, 39, 40	
Thyroid	36	36	63	37
Uterine				68

*Numbers correspond to works cited.
